# Evaluation of changes in the upper airway after Twin Block treatment in patients with Class II malocclusion

**DOI:** 10.1002/cre2.180

**Published:** 2019-03-18

**Authors:** Inmaculada Entrenas, Elena González‐Chamorro, Covadonga Álvarez‐Abad, Juan Muriel, Iván Menéndez‐Díaz, Teresa Cobo

**Affiliations:** ^1^ Orthodontics Division Universidad de Oviedo. Instituto Asturiano de Odontologia Oviedo Spain; ^2^ Orthodontics Division Universidad de Oviedo, Surgery and Medical‐Surgical Specialities, Instituto Asturiano de Odontologia Oviedo Spain; ^3^ Diagnostic Imaging Division Universidad de Oviedo Instituto Asturiano de Odontologia Oviedo Spain

**Keywords:** Class II malocclusion, mandibular hypoplasia, sleep breathing disorders, twin block, upper airway

## Abstract

The purpose of this prospective case control study is to describe in growing patients with mandibular hypoplasia, treatment outcomes following functional therapy in terms of volumetric changes in nasopharynx and oropharynx, that is, upper and lower pharynx. We recruited 60 study participants aged between 8 and 12 years having mandibular Class II malocclusion and a reduced upper airway (UA) size, as determined by McNamara cephalometric analyses. Forty patients received Twin Block treatment, whereas the remaining 20 patients did not receive treatment, thus constituting the control group. The control group included patients who did not start treatment after their first visit but returned for a consultation one or 2 years later. All patients underwent an initial teleradiography examination of the skull and a final teleradiography examination to measure changes using McNamara cephalometric analysis of the UA. Pretreatment and posttreatment changes were assessed using Student's *t* test for independent samples with a significance level of 0.05. Both anatomical structures analyzed—the upper pharynx (nasopharynx) and lower pharynx (oropharynx)—showed significant increases after treatment regardless of whether the patients were boys or girls. The controls showed a decrease in UA size on average after approximately 2 years of growth. A clear relationship exists between the mandibular advancement achieved with TB treatment and an increased UA size. Therefore, the appliance is considered suitable for improving the respiratory quality of growing patients with a decreased UA size.

## 1 INTRODUCTION

Respiratory sleep disorders (RSDs) in childhood, ranging from snoring to clinical manifestations of sleep apnoea‐hypopnoea syndrome (SAHS), are a common problems (Asensi et al., 2008; Gonzalo, 2013; Molina, 2011). Different reports suggest that the prevalence of SAHS is at least 2%, and affected patients may suffer long‐term adverse effects (Alonso‐Álvarez et al., 2012; Armalaite & Lopatiene, 2016; Molina, 2011). Although the first clinical description of SAHS dates back to 1892, Guilleminault reported on children diagnosed with SAHS by polysomnography (Molina, 2011), which is currently the diagnostic technique of choice (Alonso‐Álvarez et al., 2012; Asensi et al., 2008; del Sueño, 2005; Gonzalo, 2013; Menéndez, Bravo, & Zaldívar, 2014; Millán & Reyes, 2011), for the first time in 1976. From an aetiological perspective, tonsillar or adenoidal hypertrophy is the main cause of this syndrome in pediatric patients, and tonsillectomy or adenoidectomy is the treatment of choice (Gonzalo, 2013; Molina, 2011; Sockrider, Rosen, Farber, Rowley, & Lareau, 2009), respectively, but such patients do not always respond satisfactorily to treatment (Alonso‐Álvarez et al., 2012; Mosovich, Ontivero, Beskow, Fernández, & Vallejos, 2011; Zhang, He, & Ngan, 2013).

From a dental perspective, the upper airway (UA) has received increasing attention in orthodontics (Elfeky & Fayed, 2015; Li et al., 2014). The anatomy and function of the nasopharyngeal airways are directly associated with craniofacial development (Ali, Shaikh, & Fida, 2015a; Pavoni, Lombardo, Franchi, Lione, & Cozza, 2017). Due to this close relationship, mutual interaction is expected to occur between pharyngeal structures and the dentofacial pattern, validating the increasing interest among the orthodontic community (Pavoni et al., 2017). The indication to treat the cause of obstruction should be established by a pediatrician, otolaryngologist, or allergist, although orthodontics and dentofacial orthopedics can also improve such obstructions; therefore, interdisciplinary coordination is essential (Morales & Varela, 2017). Malocclusion and other dentofacial abnormalities can also cause SAHS, with mandibular retrognathism as one of the most important risk factors in children (Ali, Shaikh, & Fida, 2015b; Jena, Singh, & Utreja, 2013; Pupneja, Utreja, Singh, Aggarwal, & Jena, 2015). Furthermore, patients with RSDs often exhibit a retracted position of the jaw in relation to the skull base (Caridi & Galluccio, 2013; Ghodke, Utreja, Singh, & Jena, 2014; Jena et al., 2013; Maspero, Giannini, Galbiati, Kairyte, & Farronato, 2015; More et al., 2011; Pavoni et al., 2017).

The use of functional appliances to correct mandibular retrognathia is very common among orthodontists and is increasingly recommended as a treatment option for obstructive sleep apnoea (OSA; Abdelkarim, 2012; Ali et al., 2015a, 2015b; Caridi & Galluccio, 2013; Elfeky & Fayed, 2015; Maspero et al., 2015; Zhang et al., 2013). Previous studies have reported improvements in UA dimensions after functional therapy (Elfeky & Fayed, 2015; Ghodke et al., 2014; Maspero et al., 2015; Pavoni et al., 2017), with Twin Block (TB) being one of the most common and popular functional appliances due to its effectiveness in correcting skeletal Class II malocclusion (Abdelkarim, 2012; Al‐Anezi, 2011; Brunharo, Quintão, Almeida, Motta, & Barreto, 2011; Burhan & Nawaya, 2015; Elfeky & Fayed, 2015; Ghodke et al., 2014; Li et al., 2016; Schaefer, McNamara, Franchi, & Baccetti, 2004; Sharma, Sachdev, Singla, & Kirtaniya, 2012; SuchitaTarvade, Yamyar, Choudhari, & Biday, 2014; Verma, Tandon, Nagar, Singh, & Singh, 2012; Zhang et al., 2013).

This study evaluates the changes produced in the UA after TB treatment in growing patients with mandibular hypoplasia and examines respiratory improvement in patients with clinical manifestations that may predict SAHS.

## 2 MATERIALS AND METHODS

This prospective case–control study was conducted with 60 patients from the Asturian Institute of Dentistry (IAO for its acronym in Spanish), including 30 children and 30 girls aged between 8 and 12 years with a decreased UA size and mandibular Class II malocclusion.

The treatment group consisted of 40 patients with mesofacial and brachyfacial growth patterns treated with functional TB appliances, including 20 children and 20 girls. Patients with a very vertical pattern and a tendency towards an open anterior bite were excluded because although one benefit of TB appliances is control of the vertical dimension (without trimming bite blocks to avoid favoring posterior tooth extrusion; Quintero & Mariaca, 2013), another type of functional appliance is better suited for these patients. With TB treatment, vertical development is achieved by gradually trimming the bite blocks, favoring posterior tooth extrusion, and leveling the occlusal plane (Ysla, Manso, Laffite, López, & Carrera, 2005).

The remaining 20 patients formed the control group, which included patients who did not start treatment after their first visit but returned for a consultation one or 2 years later.

The TB appliance is a functional device used for early treatment of children with Class II malocclusion that advances the jaw and stimulates jaw growth (Burhan & Nawaya, 2015; Clark, 2015; Sharma et al., 2012; Ysla et al., 2005). The appliance was developed by William J Clark in 1970 in Scotland and is currently one of the most common and popular functional appliances due to its effectiveness in correcting skeletal Class II malocclusion (Abdelkarim, 2012; Al‐Anezi, 2011; Brunharo et al., 2011; Burhan & Nawaya, 2015; Elfeky & Fayed, 2015; Ghodke et al., 2014; Li et al., 2016; Schaefer et al., 2004; Sharma et al., 2012; SuchitaTarvade et al., 2014; Verma et al., 2012; Zhang et al., 2013). The appliance is well accepted by patients and can produce rapid changes (Al‐Anezi, 2011; Elfeky & Fayed, 2015; Li et al., 2014; Li et al., 2016; Quintero & Mariaca, 2013; Zhang et al., 2013). The appliance should be worn 24 hours a day (Clark, 2015; Quintero & Mariaca, 2013; Ysla et al., 2005), although use of the appliance for approximately 14–18 hours per day also yields positive effects after 12–18 months of treatment. However, treatment should be ideally initiated before or during peak growth to produce more favorable results (Quintero & Mariaca, 2013; Saldarriaga‐Valencia, Alvarez‐Varela, & Botero‐Mariaca, 2013). The appliance should be retained—either the TB itself or a Hawley plate with an advancement splint—until the end of growth to ensure long‐term stability.

Lateral teleradiography of the skull has been the main method to assess the effectiveness of the TB device and evaluate the changes produced in the UA after treatment.

**Figure 1:**
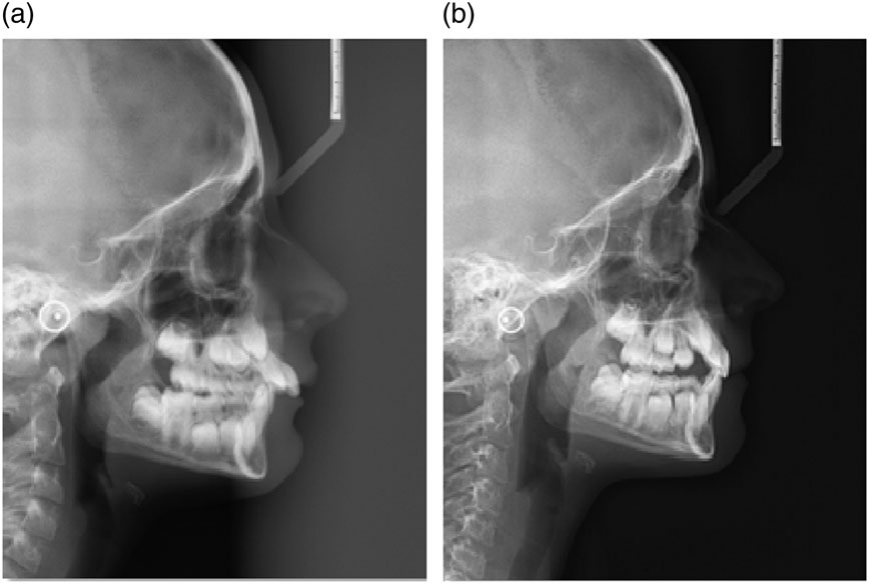
(a) Lateral teleradiography of the skull before Twin Block treatment. (b) Lateral teleradiography of the skull 18 months after Twin Block treatment. An increase in the upper airway is observed

**Figure 2:**
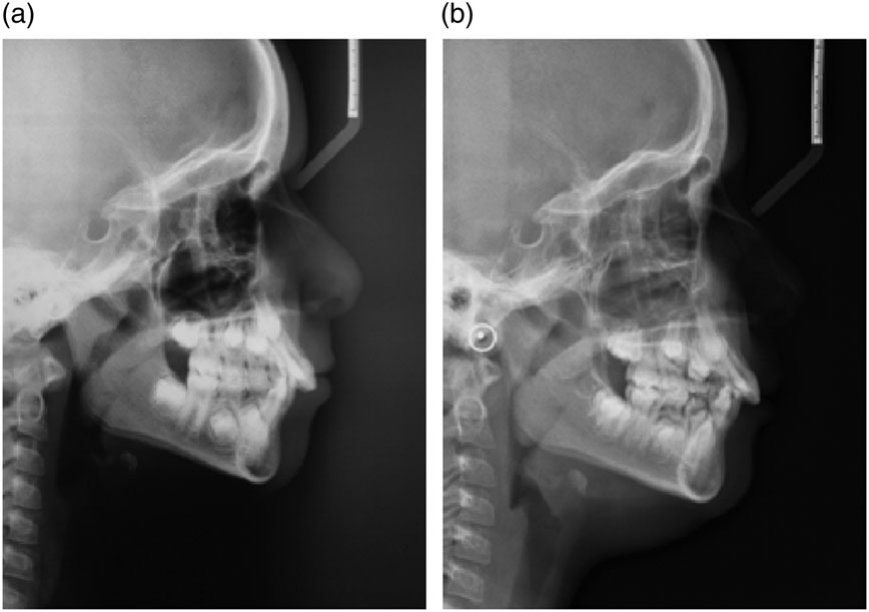
(a) Lateral teleradiography of the skull at baseline. (b) Lateral teleradiography of the skull after 2 years of growth without treatment. A decrease in the upper airway and an increase in the tonsils are observed

Both the treatment group (Figure 1) and the control group (Figure 2) underwent an initial lateral teleradiography exam of the skull and a final exam. Pretreatment and posttreatment radiographs were taken according to the manufacturer's protocol and by the same operator. The patients were placed in a natural head position, and their teeth were in maximal intercuspation with the lips and tongue at rest. They were asked not to swallow and not to move their heads or tongues.

**Figure 3:**
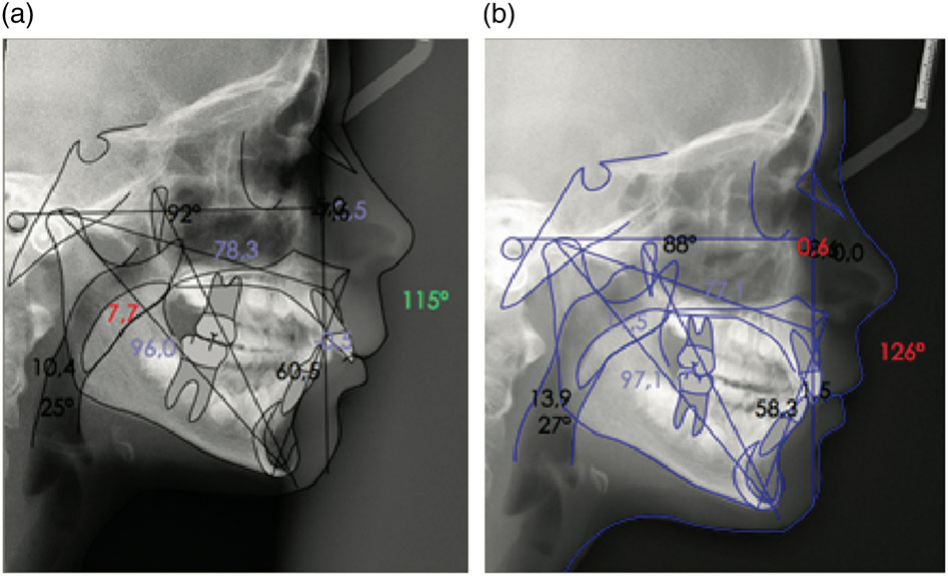
(a) McNamara analysis of the upper airway in a patient before Twin Block treatment. (b) McNamara analysis of the upper airway in the patient 18 months after Twin Block treatment. An increase in the upper airway is observed

To measure the UA and assess its patency, McNamara analysis has been used (Figure 3). Two anatomical structures are measured to identify alterations in the respiratory airways:

- The upper pharynx (nasopharynx) is defined as the region between the most posterior part of the soft palate and the nearest point on the posterior pharyngeal wall, which reflects the diameter of the nasopharynx. A larger upper pharynx ​​allows greater ventilation, whereas a smaller upper pharynx ​​indicates narrowing and a less patent airway (McNamara, 1984)
- The lower pharynx (oropharynx) is defined as the region between the intersection of the posterior border of the tongue, the lower edge of the jaw, and the point closest on the posterior pharyngeal wall, which reflects the diameter of the oropharynx. A larger lower pharynx ​indicates greater patency, whereas a smaller lower pharynx ​​indicates narrowing and poorer ventilation (McNamara, 1984).

Nemoceph cephalometric analysis software was used for radiograph tracing.

The studies and procedures were approved by the Research Ethics Committee of the Instituto Asturiano de Odontología (IAO), in the meeting held the day 10th of February 2015, with reference number Ref. IAO‐15‐057. This body analyzes problems and ethical values ​​related to the work of IAO researchers in line with current legislation.

## 3 RESULTS

### 3.1 Descriptive analysis

Of the 60 patients included in this study, 40 (66.67%) constituted the experimental group, and 20 (33.3%) constituted the control group. In all patients, the upper pharynx and inferior pharynx from the McNamara cephalometric analysis of the UA were measured according to the initial (T1) and final (T2) lateral teleradiography exams of the skull (Table 1).

**Table 1:** Patients included in this study

Age								
	n	Boys	Girls	8	9	10	11	12
Control group	20	10	10	9	6	2	1	2
Experimental group	40	10	10	6	11	10	10	3

### 3.2 Control group

The control group consisted of 10 boys and 10 girls who did not start treatment after their first visit (T1) because the parents of these patients did not aesthetically appreciate Class II malocclusion. They returned for a consultation one or 2 years later (T2) because it became evident after this time the worsening of the malocclusion. Note that the initial data of the oropharynx differ from the experimental group because in these patients was not as reduced as in patients treated with Twin Block (Table 2).

**Table 2:** Summary control group

(%)								
	n	Average	SD	0	25	50	75	100
Initial upper pharynx	40	7.96	1.98	4.10	6.80	7.65	9.25	11.60
Final upper pharynx	40	7.22	2.10	3.70	6.07	6.75	8.98	11.00
Initial lower pharynx	40	10.56	2.37	6.60	9.00	10.30	11.95	16.70
Final lower pharynx	40	9.39	2.43	5.00	7.57	9.25	10.98	13.70

#### 3.2.1 Initial upper pharynx (nasopharynx) measurements (control group)

Twenty valid cases were available for baseline upper pharynx measurements. The average value was 7.96 units with a standard deviation of 1.98, and the median value was 7.65 units.

#### 3.2.2 Final upper pharynx (nasopharynx) measurements (control group)

Twenty valid cases were available for final upper pharynx measurements. The average value was 7.22 units with a standard deviation of 2.1, and the median value was 6.75 units.

#### 3.2.3 Initial lower pharynx (oropharynx) measurements (control group)

Twenty valid cases were available for baseline lower pharynx measurements. The average value was 10.56 units with a standard deviation of 2.37, and the median value was 10.3 units.

#### 3.2.4 Final lower pharynx (oropharynx) measurements (control group)

Twenty valid cases were available for final lower pharynx measurements. The average value was 9.39 units with a standard deviation of 2.43, and the median value was 9.25 units.

### 3.3 Experimental group

**Figure 4:**
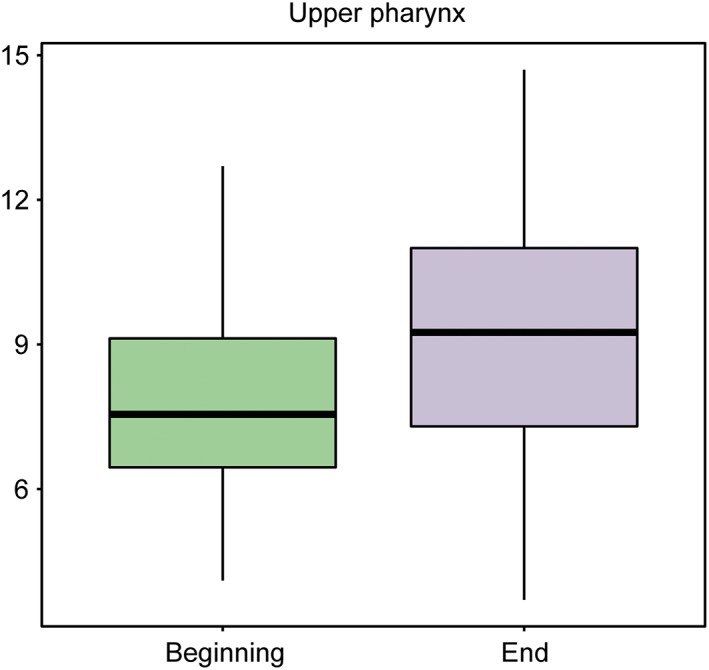
Box plot representing the upper pharynx at baseline and at the end of the study in the treatment group

**Figure 5:**
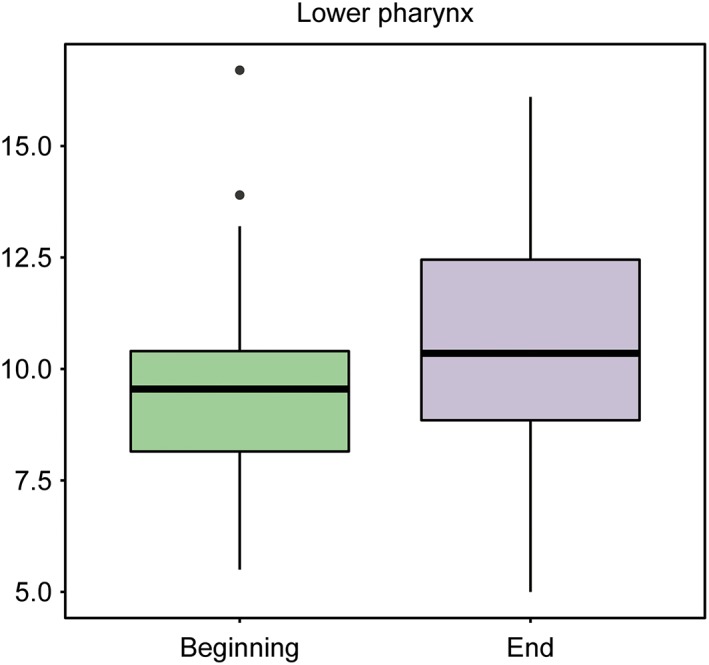
Box plot representing the lower pharynx at baseline and at the end of the study in the treatment group

The experimental group consisted of 20 boys and 20 girls. These patients were evaluated at the start of treatment, T1, and after 12–18 months of TB treatment, T2 (Figures 4 and 5; Table 3).

**Table 3:** Summary experimental group

(%)								
	n	Average	SD	0	25	50	75	100
Initial upper pharynx	40	7.88	1.94	5.00	6.20	7.55	9.10	12.70
Final upper pharynx	40	10.17	1.95	6.20	8.97	9.60	11.2	14.70
Initial lower pharynx	40	8.93	1.82	5.50	7.45	9.35	10.00	12.90
Final lower pharynx	40	11.04	2.27	6.80	9.20	10.55	13.12	16.10

#### 3.3.1 Initial upper pharynx (nasopharynx)

Forty valid cases were available for baseline upper pharynx measurements. The average value was 7.88 units with a standard deviation of 1.94, and the median value was 7.55 units.

#### 3.3.2 Final upper pharynx (nasopharynx)

Forty valid cases were available for final upper pharynx measurements. The average value was 10.17 units with a standard deviation of 1.95, and the median value was 9.6 units.

#### 3.3.3 Initial lower pharynx (oropharynx)

Forty valid cases were available for baseline lower pharynx measurements. The average value was 8.93 units with a standard deviation of 1.82, and the median value was 9.35 units.

#### 3.3.4 Final lower pharynx (oropharynx)

Forty valid cases were available for final lower pharynx measurements. The average value was 11.04 units with a standard deviation of 2.27, and the median value was 10.55 units.

### 3.4 Comparison of T1–T2

#### 3.4.1 Behavior of the upper pharynx (nasopharynx [mm]) in the different groups (control and experimental groups)

To determine whether the behavior of the upper pharynx differed between the two groups, several comparisons were performed, which are detailed below.

Because the hypothesis of normality was not rejected for all modalities (Shapiro–Wilk test, control group, *p* value = 0.21; experimental group, *p* value = 0.05) and the hypothesis of equality of the two population variances was not rejected (variance test F, *p* value = 0.37), the hypothesis of equality of the population means was rejected (Student's *t* test, *p* value < 0.001).

**Figure 6:**
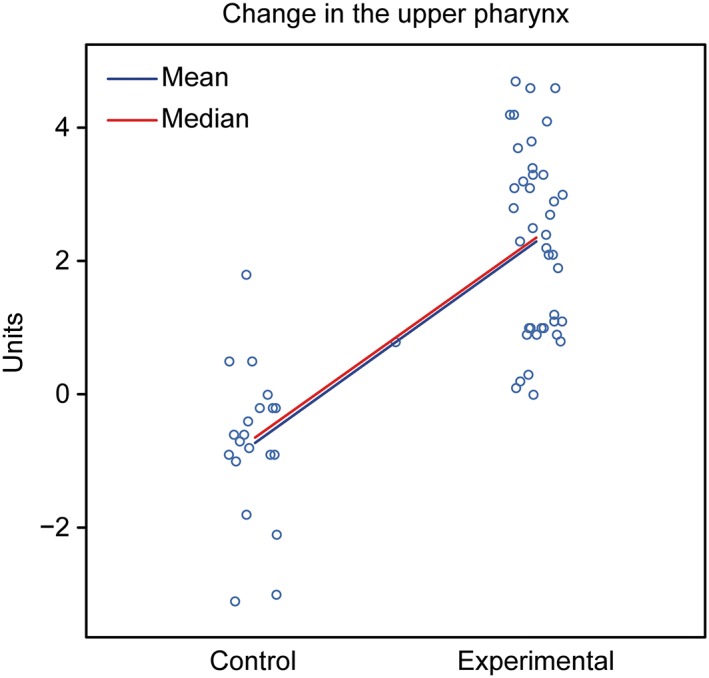
Graphical representation of the comparison of T1–T2 in the upper pharynx between the control and experimental groups

Assessment: The groups showed different behaviors. The control group showed a tendency for a decreased nasopharynx, whereas the experimental group exhibited a tendency for an increased nasopharynx (Figure 6; Table 4).

**Table 4:** Summary upper pharynx (T1–T2) and lower pharynx (T1–T2)

	Control			Experimental			
	Initial	Final	Dif final‐initial	Initial	Final	Dif final‐initial	P value
Upper Pharynx	7.96 + −1.98	7.22 + −2.10	−0.73 + −1.14	7.88 + −1.94	10.17 + −1.95	2.29 + −1.39	<0.001
Lower Pharynx	10.56 + −2.37	9.39 + −2.43	−1.18 + −1.68	8.93 + −1.82	11.04 + −2.27	2.10 + −1.78	<0.001

#### 3.4.2 Behavior of the lower pharynx (oropharynx [mm]) in the different groups (control and experimental groups)

To determine whether the behavior of the lower pharynx differed between the two groups, several comparisons were performed, which are detailed below.

Because the hypothesis of normality was not rejected for all modalities (Shapiro–Wilk test, control group, *p* value = 0.39; experimental group, *p* value = 0.08) and the hypothesis of equality of the two population variances was not rejected (variance test *F*, *p* value = 0.79), the hypothesis of equality of the population means was rejected (Student's *t* test, *p* value < 0.001).

**Figure 7:**
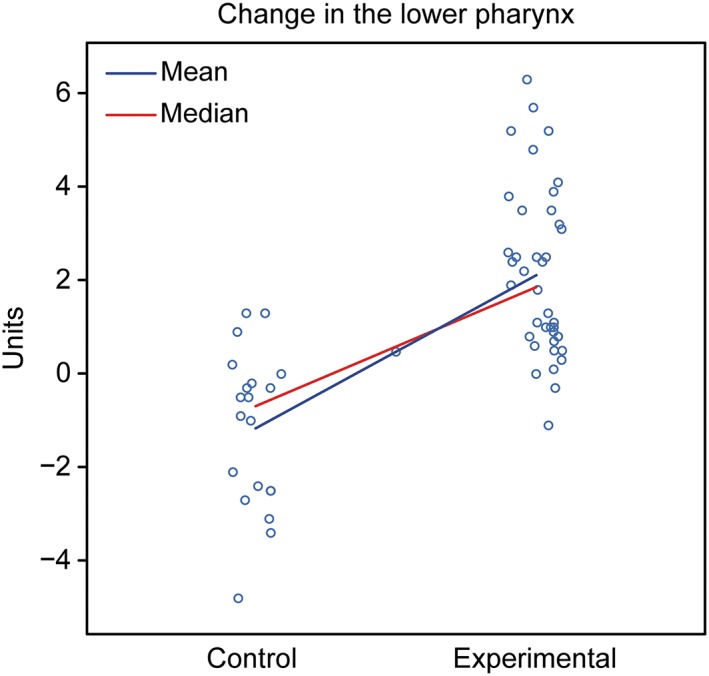
Graphical representation of the comparison of T1–T2 in the lower pharynx between the control and experimental groups

Assessment: The groups showed different behaviors. The control group showed a tendency for a decreased oropharynx, whereas the experimental group exhibited a tendency for an increased oropharynx (Figure 7).

### 3.5 Comparison of T1–T2 in girls

#### 3.5.1 Behavior of the upper pharynx (nasopharynx [mm]) in the different groups (control and experimental groups)

To determine whether the behavior of the upper pharynx differed between the two groups, several comparisons were performed, which are detailed below.

Because the hypothesis of normality was not rejected for all modalities (Shapiro–Wilk test, control group, *p* value = 0.206; experimental group, *p* value = 0.166) and the hypothesis of equality of the two population variances was not rejected (variance test *F*, *p* value = 0.383), the hypothesis of equality of the population means was rejected (Student's *t* test, *p* value < 0.001).

**Figure 8:**
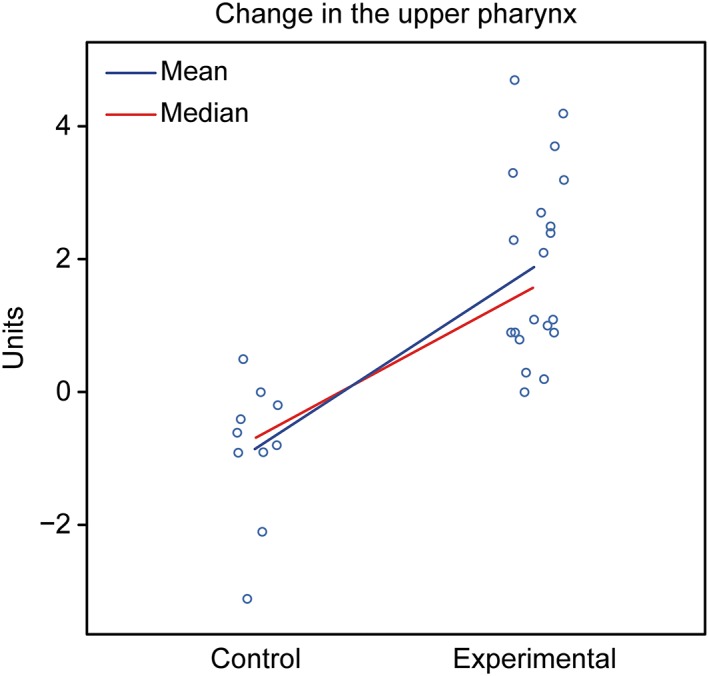
Graphical representation of the comparison of T1–T2 in the upper pharynx in girls between the control and experimental groups

Assessment: The groups showed different behaviors. The control group showed a tendency for a decreased nasopharynx, whereas the experimental group exhibited a tendency for an increased nasopharynx (Figure 8).

#### 3.5.2 Behavior of the lower pharynx (oropharynx [mm]) in the different groups (control and experimental)

To determine whether the behavior of the lower pharynx differed between the two groups, several comparisons were performed, which are detailed below.

Because the hypothesis of normality was not rejected for all modalities (Shapiro–Wilk test, control group, *p* value = 0.296; experimental group, *p* value = 0.079) and the hypothesis of equality of the two population variances was not rejected (variance test *F*, *p* value = 0.486), the hypothesis of equality of the population means was rejected (Student's *t* test, *p* value < 0.001).

**Figure 9:**
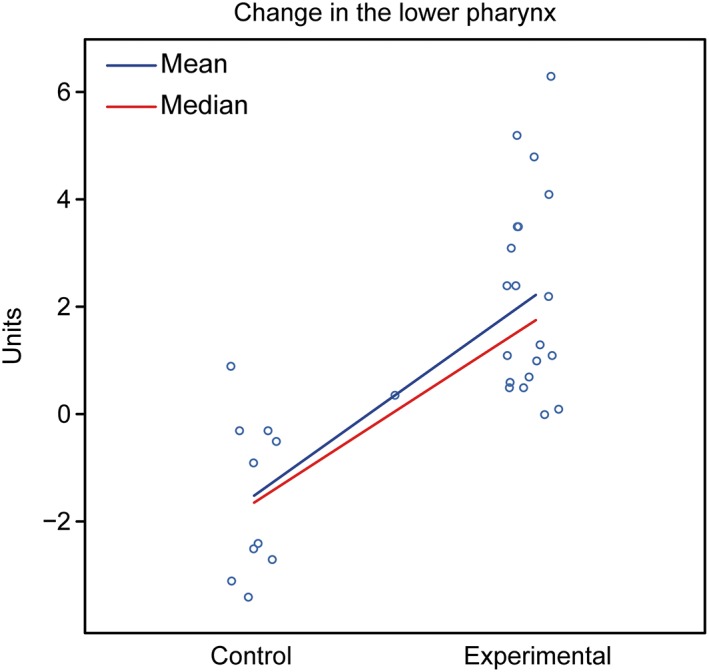
Graphical representation of the comparison of T1–T2 in the lower pharynx in girls between the control and experimental groups

Assessment: The groups showed different behaviors. The control group showed a tendency for a decreased oropharynx, whereas the experimental group exhibited a tendency for an increased oropharynx (Figure 9).

### 3.6 Comparison of T1–T2 in boys

#### 3.6.1 Behavior of the upper pharynx (nasopharynx [mm]) in the different groups (control and experimental groups)

To determine whether the behavior of the upper pharynx differed between the two groups, several comparisons were performed, which are detailed below.

Because the hypothesis of normality was not rejected for all modalities (Shapiro–Wilk test, control group, *p* value = 0.837; experimental group, *p* value = 0.387) and the hypothesis of equality of the two population variances was not rejected (variance test *F*, *p* value = 0.981), the hypothesis of equality of population means is rejected (Student's *t* test, *p* value < 0.001).

**Figure 10:**
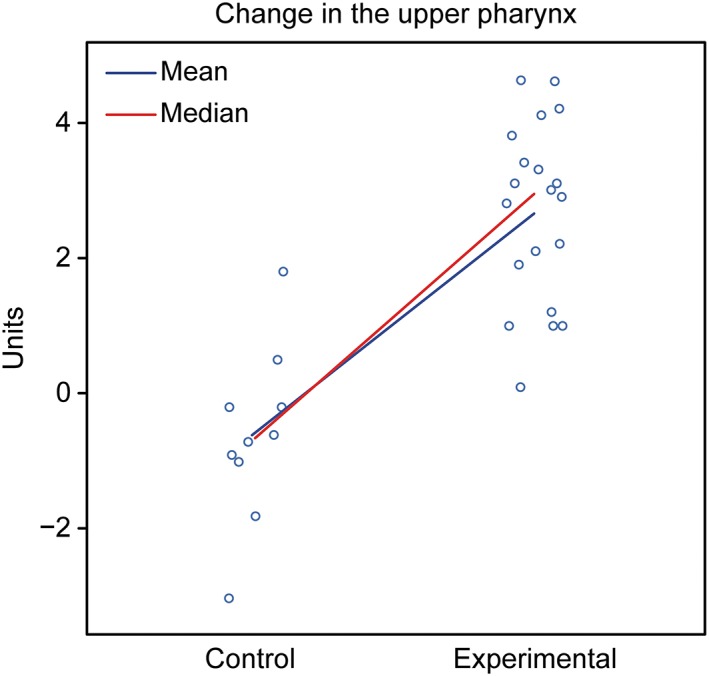
Graphical representation of the comparison of T1–T2 in the upper pharynx in boys between the control and experimental groups

Assessment: The groups showed different behaviors. The control group showed a tendency for a decreased nasopharynx, whereas the experimental group exhibited a tendency for an increased nasopharynx (Figure 10).

#### 3.6.2 Behavior of the lower pharynx (oropharynx [mm]) in the different groups (control and experimental groups)

To determine whether the behavior of the lower pharynx differed between the two groups, several comparisons were performed, which are detailed below.

Because the hypothesis of normality was not rejected for all modalities (Shapiro–Wilk test, control group, *p* value = 0.336; experimental group, *p* value = 0.577) and the hypothesis of equality of the two population variances was not rejected (variance test *F*, *p* value = 0.775), the hypothesis of equality of the population means was rejected (Student's *t* test, *p* value < 0.001).

**Figure 11:**
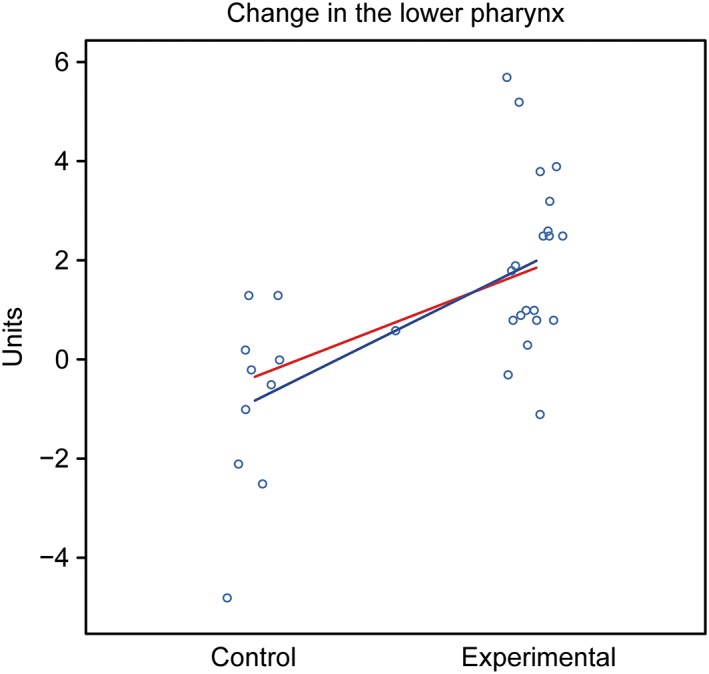
Graphical representation of the comparison of T1–T2 in the upper pharynx in boys between the control and experimental groups

Assessment: The groups showed different behaviors. The control group showed a tendency for a decreased oropharynx, whereas the experimental group exhibited a tendency for an increased oropharynx (Figure 11).

## 4 DISCUSSION

This study shows that correction of mandibular retrognathism with a TB appliance in growing patients not only improves the facial profile and intermaxillary relationship but also increases UA dimensions (Ali et al., 2015b; Roque‐Torres, Meneses‐López, Bóscolo, de Almeida, & Neto, 2015), thus reducing the risk of future respiratory problems (Verma et al., 2012) and representing a suitable oral appliance to treat children with SAHS (Caridi & Galluccio, 2013; Zhang et al., 2013). However, few studies have shown the long‐term effectiveness of TB appliances; therefore, their permanency remains to be determined.

Similarly, the most important limitation of this study is that the main diagnostic method was lateral teleradiography of the skull, which provides a two‐dimensional (2D) representation, but the UA is a 3D space, limiting the accuracy of the technique because 2D images only show the anteroposterior dimension in the sagittal plane rather than a complete view (Abdelkarim, 2012; Elfeky & Fayed, 2015; Li et al., 2014). However, lateral teleradiography of the skull is commonly used in routine clinical practice due to its relative simplicity, accessibility, low cost, and low radiation exposure (Feng et al., 2015; Rojas, Corvalán, Messen, & Sandoval, 2017; Santamaria‐Villegas, Manrique‐Hernandez, Alvarez‐Varela, & Restrepo‐Serna, 2017). Teleradiography remains a valuable diagnostic tool for evaluating the airways (Ali et al., 2015b; Elfeky & Fayed, 2015; Ghodke et al., 2014; Jena et al., 2013; Pavoni et al., 2017) and can be used to predict OSA (Armalaite & Lopatiene, 2016). Furthermore, this method has been shown to provide reliable linear measurements and is a valid tool for measuring the dimensions of the nasopharyngeal and retropalatal regions. Teleradiography is a highly reproducible examination using the natural head position of the patient when performed properly (Rojas et al., 2017).

Radiographic computer tomography (CT) provides a more accurate estimate of the UA volume and more detail compared with teleradiography (Abdelkarim, 2012; Iwasaki et al., 2014; Li et al., 2016; Maspero et al., 2015). However, the patient, and in this case the growing child, becomes exposed to higher radiation contrary to the ALARA principle and is difficult to justify from a research ethics perspective (Ali et al., 2015b; Ghodke et al., 2014).

Evidence‐based data on the radiation dose to acquire CBCT images are severely lacking, with some authors reporting lower radiation compared with conventional CT (Elfeky & Fayed, 2015). The effective dose of CBCT used for orthodontic exams is also concerning, especially because patients start orthodontic treatment in childhood; therefore, the clinical benefits must be weighed against the potential risk of radiation (Evans et al., 2013; Roque‐Torres et al., 2015).

Three‐dimensional cephalometry is one of the significant advantages that this new technology can provide; however, considerable time is required before a practical and useful 3D analysis method based on new research becomes available (Roque‐Torres et al., 2015).

During the last decade, the number of publications related to CBCT in the literature has increased significantly. This technology has been incorporated into specific applications in orthodontics for diagnosis and treatment planning in adult and pediatric patients. CBCT images provide two unique features for orthodontic practice. First, numerous linear projections (e.g., lateral cephalometric images) or flat curves (e.g., panoramic images) currently used in orthodontic diagnosis, cephalometric analysis, and treatment planning can be derived from a single CBCT scan, providing greater clinical efficiency. Second, most importantly, CBCT data can be reconstructed to provide unique images previously unavailable in orthodontic practice (Evans et al., 2013).

Although CBCT can provide volumetric measurement of the airways, no studies have demonstrated that it can be used as an accurate tool for diagnosing OSA (Roque‐Torres et al., 2015).

## 5 CONCLUSIONS

A significant increase in UA size was observed in both the nasopharynx and oropharynx after early treatment with TB appliances in patients with mandibular Class II malocclusion. The effectiveness of TB treatment was demonstrated in patients with clinical manifestations suggestive of SAHS, mouth breathing, and/or snoring as all patients showed improved respiratory quality.

Patients with mandibular Class II malocclusion show a decrease in UA size with growth and may therefore become future SAHS patients if not treated with functional appliances.

TB devices are some of the most common and popular functional appliances due to their effectiveness in skeletal Class II correction, thus improving the facial profile. In addition, these devices may be effective for treating children with RSDs and mandibular retrognathia, thus decreasing the risk of SAHS development in adulthood.

Whenever CBCT is performed in orthodontic practice, the clinical benefits to the patient must be weighed against the potential risk of radiation.

Currently, more research is being conducted on the benefits of intraoral orthopedic appliances for the treatment of SAHS and other RSDs; however, few studies have demonstrated the long‐term stability of such devices.

## CONFLICTS OF INTEREST

The authors have no conflicts of interest to declare.
